# A Green Approach to Obtaining Glycerol Carbonate by Urea Glycerolysis Using Carbon-Supported Metal Oxide Catalysts

**DOI:** 10.3390/molecules28186534

**Published:** 2023-09-09

**Authors:** Karolina Ptaszyńska, Anna Malaika, Klaudia Kozigrodzka, Mieczysław Kozłowski

**Affiliations:** Faculty of Chemistry, Adam Mickiewicz University in Poznań, Uniwersytetu Poznańskiego 8, 61-614 Poznań, Poland; amalaika@amu.edu.pl (A.M.); klakoz3@st.amu.edu.pl (K.K.)

**Keywords:** glycerol valorization, carbon support, carbon fibers, glycerolysis, glycerol carbonate

## Abstract

The results of sustainable and selective synthesis of glycerol carbonate (GC) from urea and glycerol under ambient pressure using carbon-fiber-supported metal oxide catalysts are reported. Carbon fibers (CF) were prepared via a catalytic chemical vapor deposition method (CCVD) using Ni as a catalyst and liquefied petroleum gas (LPG) as a cheap carbon source. Supported metal oxide catalysts were obtained by an incipient wetness impregnation technique using Zn, Ba, Cr, and Mg nitrates. Finally, the samples were pyrolyzed and oxidized in an air flow. The obtained catalysts (10%Me_x_O_y_/CF_ox_) were tested in the reaction of urea glycerolysis at 140 °C for 6 h under atmospheric pressure, using an equimolar ratio of reagents and an inert gas flow for NH_3_ removal. Under the applied conditions, all of the prepared catalysts increased the glycerol conversion and glycerol carbonate yield compared to the blank test, and the best catalytic performance was shown by the CF_ox_-supported ZnO and MgO systems. Screening of the reaction conditions was carried out by applying ZnO/CF_ox_ as a catalyst and considering the effect of reaction temperature, molar ratio of reagents, and the mode of the inert gas flow through the reactor on the catalytic process. Finally, a maximum yield of GC of about 40%, together with a selectivity to glycerol carbonate of ~100%, was obtained within 6 h of reaction at 140 °C using a glycerol-to-urea molar ratio of 1:1 while flowing Ar through the reaction mixture. Furthermore, a positive heterogeneous catalytic effect of the CF_ox_ support on the process was noticed.

## 1. Introduction

**Figure 1:**
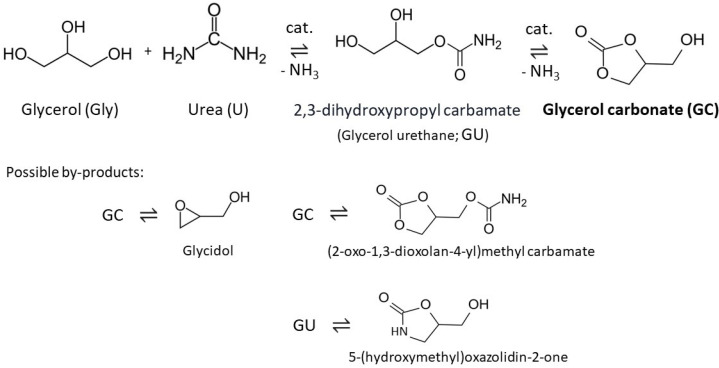
Synthesis of glycerol carbonate (GC) from glycerol and urea (based on [8,9]).

In recent years, using biomass feedstocks instead of non-renewable petrochemical resources to sustainably produce commodities and chemicals has been gaining significant importance. Glycerol (Gly), obtained in vast amounts as a by-product in biomass-based biodiesel technology, can be used as a green, renewable, readily available, and versatile feedstock for obtaining higher-value-added chemical products such as glycerol carbonate (GC), among others [1]. Glycerol carbonate (4-hydroxymethyl-1,3-dioxolan-2-one) has a wide range of industrial applications, including the production of high-performance hyperbranched polymers, coatings, adhesives, and lubricants. It is also used as a solvent, detergent, and curing agent for cement and concrete, in the lithium-ion battery industry, or in gas separation units [1,2,3]. Among possible GC synthesis routes, urea glycerolysis (Figure 1) is considered an economically attractive and environmentally benign approach [4], and can be an alternative to the conventional method of GC production using toxic phosgene [5]. According to literature reports, several reaction pathways of urea glycerolysis are possible, and the process requires acidic or basic Lewis active sites, or the presence of a bifunctional catalyst [4,5,6,7].

Urea glycerolysis can be effectively homogeneously catalyzed by various metal-based compounds. For example, Wang et al. [10] found that LaCl_3_ could give a 95.4% conversion of Gly with almost 100% selectivity to glycerol carbonate under optimized reaction conditions, i.e., 3 h, 150 °C, and 5 kPa. Park et al. [11] reported that homogeneous ZnCl_2_ yielded 80.2% of GC with 99.7% selectivity to this product when the reaction was conducted at 150 °C for 2 h under reduced pressure. In turn, Turney et al. [12] showed that polymeric monoglycerolate complexes of zinc and cobalt could give such a high glycerol conversion as 98% and a GC yield of 83% at 140 °C within 7 h.

Despite the promising results typically obtained in urea glycerolysis with homogeneous catalysts, these processes usually show serious drawbacks such as separation difficulties and catalyst unrecoverability [13]. Thus, a more practical and economical option for producing GC via urea glycerolysis is to use heterogeneous metal-based catalysts in this reaction. For example, Wang et al. [7] tested different forms of lanthanum oxide as solid catalysts for glycerolysis of urea performed at 140 °C, obtaining a very high GC yield and selectivity to glycerol carbonate of ~90 and 97%, respectively, for the best-working sample (La_2_O_3_-600). The enhanced catalytic performance of the La_2_O_3_-600 was attributed to the increased strength of Lewis basic sites, and the catalyst was reused without significant loss in its activity during recycling tests. Aresta et al. [13] obtained 80% glycerol conversion and 100% selectivity towards glycerol carbonate using ƴ-Zr phosphate. Furthermore, the authors found this catalyst to be easily recoverable and reusable in subsequent reaction cycles. An efficient reusable catalyst for urea glycerolysis was also Sn(OH)_2_, which showed 87% conversion of glycerol and 85% selectivity to GC in the reaction that lasted 4 h [14]. Interesting results were also presented by Fernandes et al. [15] who tested MgO as a catalyst for GC production under atmospheric pressure. The authors found that the sample exhibited excellent catalytic behavior, giving glycerol conversions of up to 70% with a 100% selectivity to GC within 6 h. Solid zinc-based catalysts have also been widely investigated [16,17]. For example, Zn-based mixed oxides (ZnMeO; Me = Co, Cr, and Fe) were used in urea glycerolysis performed at 140 °C under vacuum pressure. The Zn-rich mixed oxides showed better performance in the process than the Zn-poor ones, presenting 74–76% conversion of Gly and about 70–74% selectivity to GC within 3 h. However, the process occurred mainly homogeneously, which is a common problem of Zn-type catalysts [18]. On the other hand, information about the full recoverability of ZnO in properly prepared systems, e.g., with the addition of CuO, has also been reported [17].

In recent years, carbon materials such as carbon nanotubes and fibers (CNT and CNF, respectively), graphene, carbon dots (CD), hydrothermal carbons (HTC), and activated carbons (AC) have gained increasing attention in the field of catalysis. It has been found that adequately tailored carbons can be used as efficient heterogeneous catalysts for various catalytic processes, including the sustainable transformation of biomass or (bio)glycerol to value-added chemicals [19,20,21]. Carbons can also be applied as attractive supports for enzymes, metals, or metal oxide catalysts [22,23,24]. This is due to their key advantages, which include high surface area, resistance to acidic or basic media, tunable physicochemical structure, and different options for active phase immobilization [25]. Importantly, the role of carbons as supports in heterogeneous reactions is not only limited to the deposition and dispersion of the active phase on a solid sample. Carbon supports can also increase the catalyst efficiency by means of active phase–support interactions, reagent adsorption, or the presence of defects [26,27].

Various supports for the active phase have been tested in urea glycerolysis. Kondawar et al. [28] deposited Zn oxide on MCM-41, SBA-15, ZrO_2_, SiO_2_, Al_2_O_3_, and sulfated ZrO_2_ (s-ZrO_2_) using a wet impregnation method. It was found that the nature of support influenced the surface area, crystallinity, and acid/base properties of the respective catalysts. The samples varied significantly in their catalytic performances. 5%Zn/SiO_2_ was the least active, giving only a 20% conversion of glycerol. Using s-ZrO_2_ as support instead of ZrO_2_ led to a significant improvement in selectivity to GC while maintaining the conversion of Gly (~50%). Finally, the best catalytic results (X_Gly_ = 78%, S_GC_ = 98%) were shown by 5%Zn/MCM-41 presenting weak acidity; however, offering maximum dispersion of ZnO due to the high MCM-41 apparent surface area. Furthermore, the possible role of the -OH groups of the MCM-41 support was also stressed. In turn, Hammond et al. [29] evaluated the activity of gold supported on TiO_2_, commercial carbon, Nb_2_O_5_, ZnO, and MgO in urea glycerolysis. A significantly increased conversion of Gly and selectivity to GC (80% and ~70%, respectively, after 4 h) compared to the blank were obtained using MgO as support. The other supported Au catalysts exhibited moderate activity in the reaction and a rather low selectivity to GC.

Although several studies on urea glycerolysis have been performed to date, most of the reported catalysts were used under reduced pressure. In the current work, carbon fibers prepared by a facile CCVD method using a cheap carbon precursor, i.e., liquefied petroleum gas (LPG), were applied as support for different metal oxides (ZnO, BaO, Cr_2_O_3_, and MgO). These samples were tested in a solvent-free reaction of urea with glycerol under ambient pressure for the first time. Effects of the reaction parameters such as temperature, glycerol to urea molar ratio, and Ar flowing mode were also investigated.

## 2. Discussion of the Results

### 2.1. Characterization of the Samples

**Figure 2:**
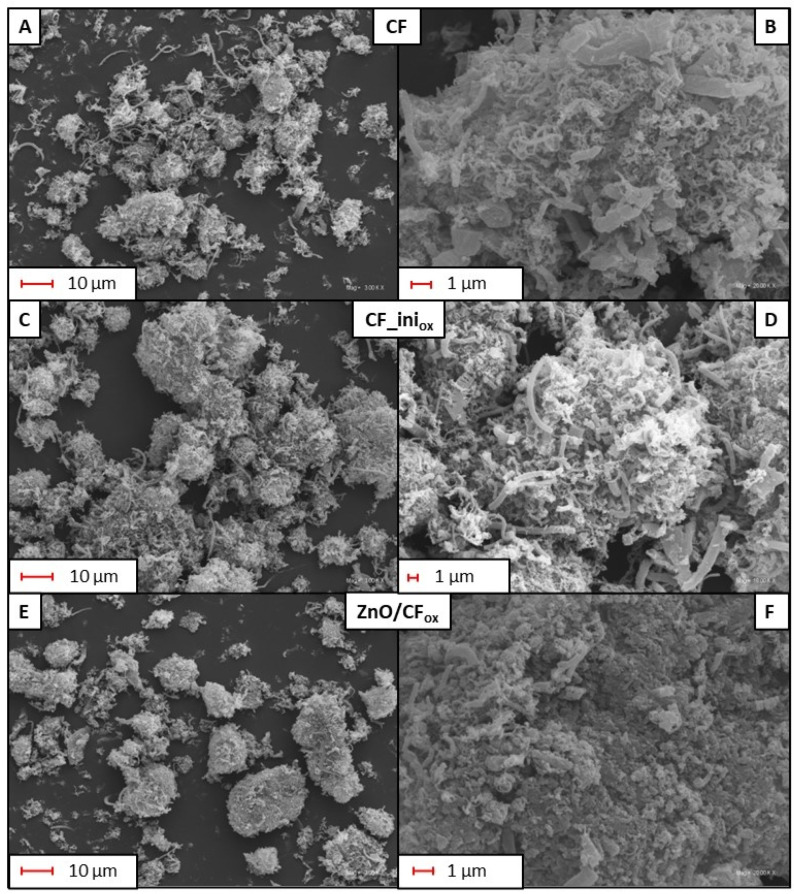
SEM micrographs obtained for the CF (**A**,**B**), CF_ini_ox_ (**C**,**D**), and a selected CF_ox_-supported catalyst (**E**,**F**) at different magnifications.

Figure 2 shows SEM micrographs of the initial CF, CF_ini_ox_, and a selected CF_ox_-supported metal oxide catalyst. As can be seen in Figure 2A, the applied CCVD method of carbon production gave a sample formed by entangled fibers organized into agglomerates of various shapes and sizes; however, single filaments could also be observed. Details of the morphology of CF are shown in Figure 2B. According to the presented image, the fibers formed agglomerates differing in their morphological features. A part of the produced structures presented low-diameter fibers that were curled and twisted, and resembled sheep’s wool. The other structures showed diameters that were several times higher and rather smooth surfaces. The visible cross-sections of the fibers suggested that they were solid inside. All the formed structures were densely packed, and no significant numbers of holes or channels could be observed between the individual filaments. The morphological features of CF_ini_ox_ were quite similar to those of CF, as can be observed by comparing Figure 2A,B with Figure 2C,D.

The morphology of the CF_ox_-supported metal oxide catalysts was significantly different from that of the CF-type samples discussed above. As presented for an exemplary material (ZnO/CF_ox_) in Figure 2E, the original carbon agglomerates were smoother, and the carbon fibers were barely visible, as the crust (most likely formed by deposited zinc oxide) covering the fibers was produced (see Figure 2F). Similar observations were also made by Arsalani et al. [30], who deposited ZnO on the surface of carbon nanotubes.

Table 1 presents the results of the textural analysis of the obtained products, i.e., CF-type samples and CF_ox_-supported metal oxides. It can be seen that the produced CF possessed a quite significant apparent surface area of 259 m^2^/g. This resulted mainly from the presence of the external surface area, i.e., the area of meso- and macropores, which was almost 187 m^2^/g. Interestingly, the total volume of pores (V_tot_) was also significant and was mostly related to the presence of spaces of meso- and macropore sizes between the agglomerates (see also Figure 2A,C), as V_micro_ was negligible. This is also in agreement with the observations from the SEM analysis (see the discussion on Figure 2), suggesting the tightly packed structure of the agglomerates. The textural parameters of CF_ini_ox_ were only slightly different from those of CF. The only exception was the V_tot_ value of CF_ini_ox_, which was reduced compared to that of CF, suggesting the efficient introduction of oxygen groups into the CF matrix during the oxidation process (see Section 3: Materials and Methods) and their location at the entrances of the existing pores [31].

**Figure 3:**
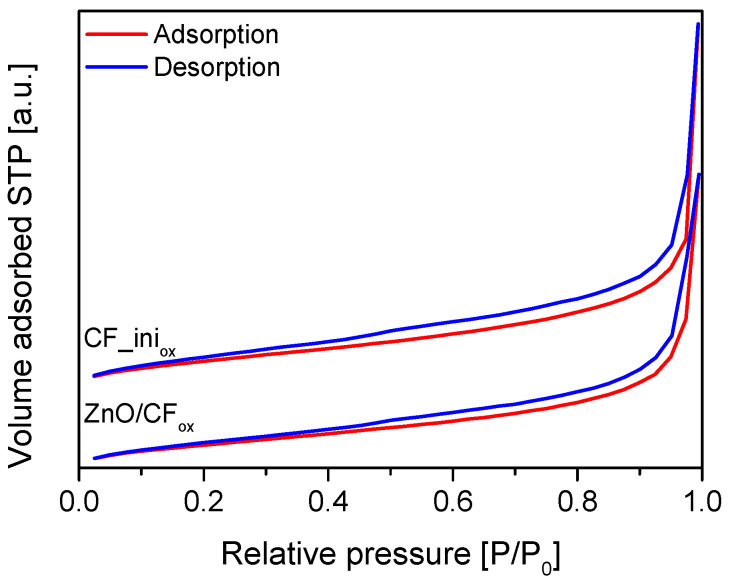
N_2_ adsorption–desorption isotherms obtained for the CF_ini_ox_ and ZnO/CF_ox_ samples.

In general, the Me_x_O_y_/CF_ox_ systems showed slightly decreased textural parameters, i.e., S_BET_, S_ext_, and V_tot_, compared to CF_ini_ox_, which confirmed the efficient loading of the respective metal oxides into the support matrix—either on its surface or in their pores [32]. This is also in accordance with the results of the SEM measurements. The mesoporous structure of the pristine CF_ini_ox_ was maintained after the deposition of metal oxides, which was suggested by a high contribution of S_ext_ to S_BET_ being achieved for the Me_x_O_y_/CF_ox_ systems, and also by the shape of the N_2_ adsorption–desorption isotherms obtained for the CF_ini_ox_ and an exemplary CF_ox_-supported sample presented in Figure 3 (IVa type acc. IUPAC classification [33]), which is typical for materials containing mesopores.

**Figure 4:**
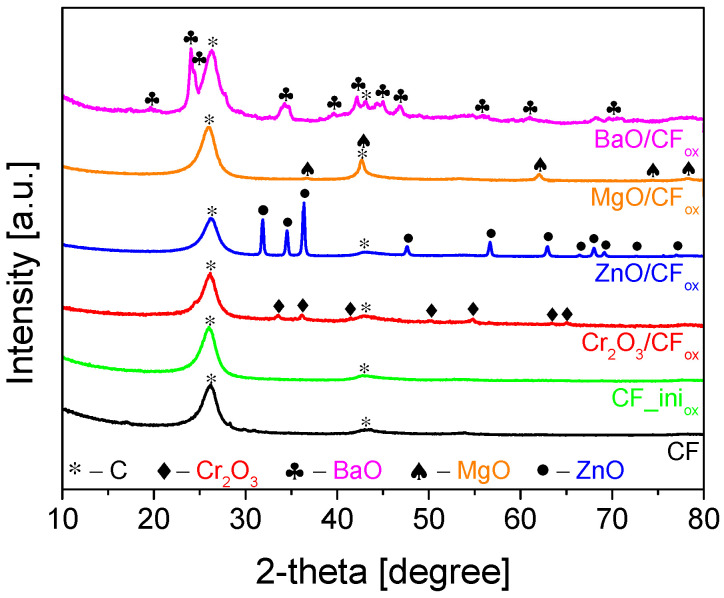
XRD patterns of CF, CF_ini_ox_, and CF_ox_-supported metal oxide samples.

The XRD diffraction patterns of the prepared samples are presented in Figure 4. The diffractograms obtained for CF and CF_ini_ox_ showed an intense peak at 2-theta of 26° and a small one at 44°. These signals are typical for graphite-like carbons and can be assigned to the C(002) and C(100) reflections of the hexagonal structure of graphite and the atomic structure of the graphene sheets, respectively [34,35,36]. No other peaks were observed in the CF and CF_ini_ox_ diffraction patterns, suggesting thorough purification of the samples from the catalyst after the CCVD process (see Section 3: Materials and Methods). The XRD diffractograms of Me_x_O_y_/CF_ox_ samples showed additional signals besides those at 26° and 44° obtained for CF-type materials, indicating the efficient loading of the respective oxides [37,38,39,40] to the carbon matrix and suggesting their crystalline forms.

**Figure 5:**
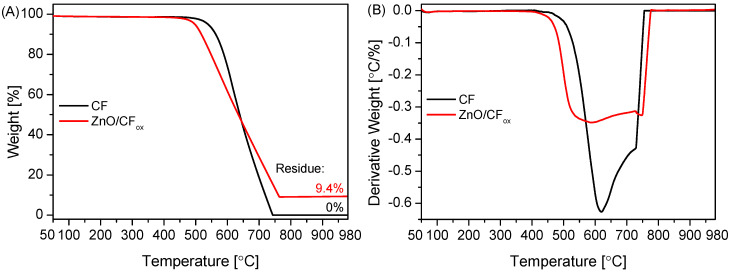
The results of (**A**) TG and (**B**) DTG analysis performed for selected samples (air flow).

Figure 5 presents the results of the TG analysis obtained for selected samples. As can be seen in Figure 5A, no significant changes in the mass of the initial CF support were observed up to about 500 °C. Rapid weight loss started at 530 °C, and the sample was completely oxidized at ~740 °C. The residue after combustion was 0%, indicating high sample purity (i.e., efficient removal of Ni catalyst after the preparation process, see Section 3: Materials and Methods) and is also in line with the results of XRD analysis (Figure 4). The DTG pattern of the initial CF (Figure 5B) resembles profiles obtained for fibrous carbons produced using the CCVD method [41]. The presence of two poorly separated peaks with minima at ~620 °C and ~740 °C indicates the existence of phases with various thermal stability, resulting from differences in the sample crystallinity, number of defects, or fiber diameters [41,42], as also suggested by the SEM results (Figure 2).

The TG profile of an exemplary metal-oxide-type sample, i.e., ZnO/CF_ox_, depicted in Figure 5A, suggested a slightly lower stability of the supported catalyst compared to the initial CF, as the weight loss of the ZnO/CF_ox_ started at ~480 °C. This shift in the onset temperature was probably related to the release of some oxygen groups present on the surface of the CF_ox_ support [43], introduced during the sample oxidation step (see Section 3: Materials and Methods). Interestingly, at a temperature of 760 °C, the carbon material was totally oxidized, and the residue after combustion was 9.4%. This residue was due to the presence of zinc oxide in the sample, and the obtained value was close to the assumed one (10 wt.%, see Section 3: Materials and Methods). The DTG pattern of ZnO/CF_ox_ in Figure 5B resembles that of CF, and the changes in the profile shape could be attributed to the formation of ZnO coating the support [44]. This also agrees well with the results of the SEM analysis presented in Figure 2F.

**Figure 6:**
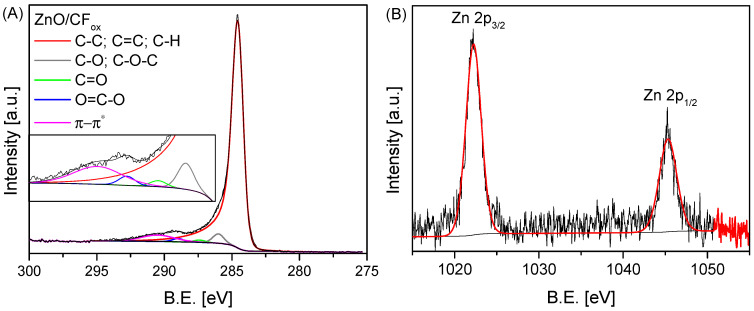
The high-resolution XPS C 1s (**A**) and Zn 2p (**B**) spectra of the ZnO/CF_ox_ sample.

The X-ray photoelectron spectroscopy (XPS) technique was used to study the surface chemistry of the ZnO/CF_ox_ sample. The achieved results are collected in Table 2 and Figure 6. The obtained data confirmed the presence of C, O, and Zn in the material. The content of elemental carbon (Table 2) was dominant, which was not surprising considering the assumed role and the amount of carbon fibers in the system. The presence of oxygen and zinc confirmed the successful loading of ZnO to the carbon matrix. The presence of oxygen could also result from the oxidation of the support during the preparation stage (see Section 3: Materials and Methods). Interestingly, the estimated zinc oxide content was 9.3%, which is also in line with the value determined by the TG analysis (see Figure 5A).

Figure 6A shows the high-resolution C 1s and Zn 2p XPS spectra obtained for the ZnO/CF_ox_ sample. As can be observed, five different carbon species were found in the C 1s spectrum—at a B.E. of ~284.5 eV (assigned to sp^3^/sp^2^ carbon), 286.0 eV (assigned to C-O/C-O-C groups), 287.4 eV (ascribed to C=O in carbonyls), 288.7 eV (ascribed to O–C=O in carboxyls), and 290.0 eV (assigned to π-π* transitions) [21]. The obtained data indicate that the CF support was successfully oxidized during the catalyst preparation stage (see Section 3: Materials and Methods). On the other hand, the Zn 2p XPS spectrum shown in Figure 6B presents the characteristic doublet peaks of Zn 2p, corresponding to the Zn^2+^ oxidation state at 1022.2 eV for Zn 2p_2/3_ and at 1045.3 eV for Zn 2p_1/2_ [45,46].

### 2.2. Catalytic Results

The catalytic activities of the prepared CF_ox_-supported samples were measured in the reaction of glycerol with urea performed under ambient pressure. For the sake of comparison, reactions without a catalyst (blank test) and in the presence of a homogeneous catalyst (ZnSO_4_) were also performed. 

**Figure 7:**
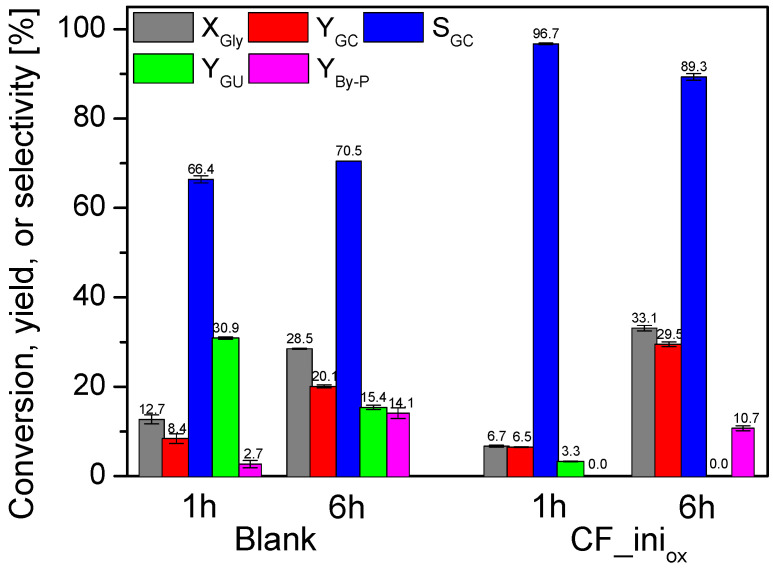
The results of urea glycerolysis performed without a catalyst (blank test) and in the presence of the CF_ini_ox_ sample; temp. = 140 °C, Gly:U molar ratio = 1:1, Ar flowing through the reactor.

To determine the influence of the support on urea glycerolysis, the CF_ini_ox_ sample was tested in the process as a catalyst on its own. Figure 7 shows the results obtained after 1 and 6 h of reaction performed over CF_ini_ox_ in comparison to the data achieved in the blank. As can be seen, under the conditions used, the reaction occurred without a catalyst; however, the glycerol conversion measured after 1 h for the blank test was quite low, i.e., about 13%. Interestingly, X_Gly_ determined for the reaction over CF_ini_ox_ was twice as small as for the blank (6.7% after 1 h). Importantly, the conversion of glycerol increased over time in both cases, reaching a higher value, i.e., of about 33% after 6 h, for the reaction applying CF_ini_ox_. Finally, the glycerol carbonate yield (Y_GC_) obtained in the process over CF_ini_ox_ was about 30%, which was almost 10% higher than the value achieved for the blank. Interestingly, the use of CF_ini_ox_ as a catalyst resulted in significant changes in the distribution of products, i.e., the selectivity to glycerol carbonate increased to about 97% after 1 h when using CF_ini_ox_, and only traces of glycerol urethane (GU; the process intermediate) were detected in the reaction. On the other hand, in the case of the blank test, S_GC_ was equal to only ~66%. Instead, a fairly high selectivity to GU, of about 31%, was achieved in the first hour of the process. Some amounts of by-products, i.e., 5-(hydroxymethyl)oxazolidin-2-one and (2-oxo-1,3-dioxolan-4-yl)methyl carbamate, were also detected (S_B-Py_ of about 3%). S_GU_ achieved for the blank decreased significantly over time, and finally, at 6 h, this parameter was about 15%. At the same time, the selectivities to glycerol carbonate (S_GC_) and by-products increased, albeit to a different extent. Finally, S_GC_ achieved at the 6th hour of the blank was only slightly higher than that obtained after the first hour of the reaction, and apparently, glycerol carbonate was mainly transformed into the by-products. In turn, in the presence of C_ini_ox_, the process seems to be much more focused on the production of glycerol carbonate, as the selectivity to by-products was reduced compared to the blank. 

The differences between the results obtained for the blank and the CF_ini_ox_ catalyst (Figure 7) most probably result from the differences in the mechanism of urea glycerolysis under non-catalytic and catalytic conditions. According to literature data [9], the crucial step of urea glycerolysis is urea splitting, which becomes the driving force for the blank process. On the other hand, the reaction performed in the presence of CF_ini_ox_ most likely occurs through a different mechanism (which is much more complex than that of homogeneous/blank reaction) and it involves various stages, i.e., diffusion of the reactants to the catalyst surface, adsorption of reagents on the catalyst active sites, chemical reaction, and desorption and diffusion of the reaction products. Moreover, in this case, the catalytic process most probably occurs due to the abundance of different oxygen groups that are present on the surface of the CF_ini_ox_ sample (see also Figure 6). It cannot be excluded that some of these groups combine with the reagents, forming quite stable bonds, and thus work as inhibition sites of the process (as the catalytic reaction is only possible when the formation of unstable intermediates between the solid catalyst and the reagents takes place). As long as these types of groups are present on the catalyst surface, the catalytic reaction is inhibited, and the so-called induction period can be obtained. This might be a reason for the lower glycerol conversion observed for CF_ini_ox_ compared to the blank. Only after the “inhibition” sites have been consumed, and thus after the induction time, can other oxygen centers work unhindered, forming unstable intermediates with the reagents; therefore, the catalytic effect can become clearly visible after a longer time. 

**Figure 8:**
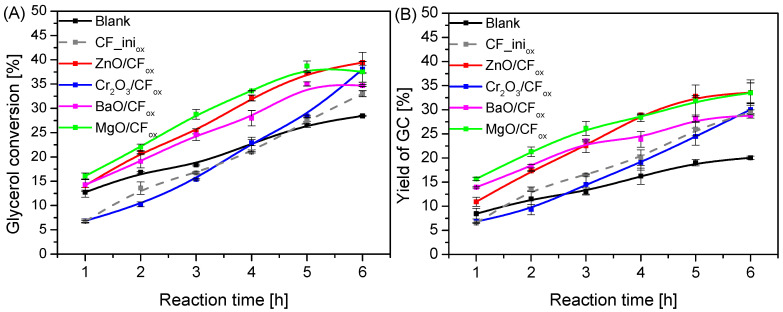
Catalytic performances of the prepared Me_x_O_y_/CF_ox_ systems in the process of urea glycerolysis versus time, expressed as glycerol conversion (**A**) and yield of GC (**B**), in comparison to the blank and the performance of the CF_ini_ox_; temp. = 140 °C, Gly:U molar ratio = 1:1, Ar flowing through the reactor.

The catalytic performances of the prepared CF_ox_-supported metal oxide catalysts in comparison to the blank and CF_ini_ox_ are shown in Figure 8. As can be seen in Figure 8A, the glycerol conversion obtained for most of the prepared catalysts after 1 h was in the range of 14–16%, which was only slightly higher than X_Gly_ achieved for the blank. The only exception was the Cr_2_O_3_/CF_ox_ sample, for which X_Gly_ measured after 1 h of reaction was only ~7%. The conversion of glycerol increased significantly over time for all of the samples, and finally, for the best-working catalyst, i.e., ZnO/CF_ox_, it was 40% after 6 h. Nevertheless, the MgO/CF_ox_ catalyst also worked effectively, which was particularly noticeable at the beginning of the process. Considering the yield of glycerol carbonate presented in Figure 8B, it can be seen that using the CF_ox_-supported systems (except for Cr_2_O_3_/CF_ox_) resulted in a quite significant increase in Y_GC_ compared to the blank, and for the most active samples, i.e., ZnO/CF_ox_ and MgO/CF_ox_, the yield of glycerol carbonate reached ~34% after 6 h of reaction. The material containing chromium oxide worked the worst in the reaction, presenting similar results to those obtained in the case of CF_ini_ox_ (other catalysts worked better than CF_ini_ox_). This suggests that the activity of the Cr_2_O_3_/CF_ox_ material resulted only from the presence of the carbon support and that the Cr_2_O_3_ phase was practically not active in the reaction. However, this conclusion assumes that CF_ini_ox_ possesses the same properties as the CF_ox_ support in the Cr_2_O_3_/CF_ox_ system. In fact, the CF_ini_ox_ is a kind of model sample; therefore, the chemical nature of these two (the oxidation of CF with supported metals most probably occurs differently than the oxidation of the pure support, see also Section 3: Materials and Methods) and their catalytic effect in the reaction may be slightly different.

Interestingly, the distribution of individual reaction products differed significantly between the blank and the samples tested, as presented in Figure S1 in the Supplementary Materials. In the case of the blank test, the selectivity to GC through the whole process was significantly lower compared to those obtained using Me_x_O_y_/CF_ox_ catalysts. On the other hand, under these conditions, the selectivity to GU was the highest. With the increasing conversion of glycerol over time (see also blank in Figure 8A), the selectivity to GU decreased. At the same time, the selectivity to glycerol carbonate was practically stable, and the by-products were formed. The use of Me_x_O_y_/CF_ox_ catalysts changed the distribution of products significantly. When using ZnO/CF_ox_, a fairly high selectivity to GU (an intermediate product) was observed in the initial hours of the process. GU was then gradually converted to glycerol carbonate, as well as some by-products. In the case of the other samples, the S_GC_ was initially very high. However, this parameter decreased slightly over time, as GC was probably further converted to by-products. 

Importantly, when considering the catalytic performances of the Me_x_O_y_/CF_ox_ systems, some differences in the samples’ activities can be observed. According to literature reports, the process of urea glycerolysis requires the presence of both acidic and basic active sites. Furthermore, ensuring the appropriate ratio of acidic to basic centers is crucial for this reaction [16,47,48]. For example, Kondawar et al. [32] tested different supported Zn catalysts in urea glycerolysis and ascribed the promising performance of the most active sample to the appropriate balance of both acidic and basic sites in the catalytic system used. At the same time, the authors found that a decreased acidic-to-basic active site (A/B) ratio in the catalysts negatively affected the selectivity to glycerol carbonate, promoting the further reaction of GC towards by-products. Interestingly, Nguyen-Phu et al. [16] proved that urea glycerolysis with the use of solid catalysts can proceed by several different mechanisms. The authors reported that the reaction can occur both in a homogeneous or heterogeneous way or even according to both variants simultaneously (with a partial dissolution of the active phase) depending on the catalyst. Moreover, they proved that the process can occur through a direct reaction of urea with glycerol or by the formation of an intermediate metal isocyanate (Me NCO) complex. According to the authors, all these factors affected the reaction rate and catalytic performances of the tested samples. Therefore, it can be supposed that in our case, the differences in the catalytic activities of the CF_ox_-supported systems were also related to the differences in the catalyst nature (e.g., acidic to basic site ratio) and to the differences (dissimilarities) in the reaction mechanism over various metal oxides used. 

**Figure 9:**
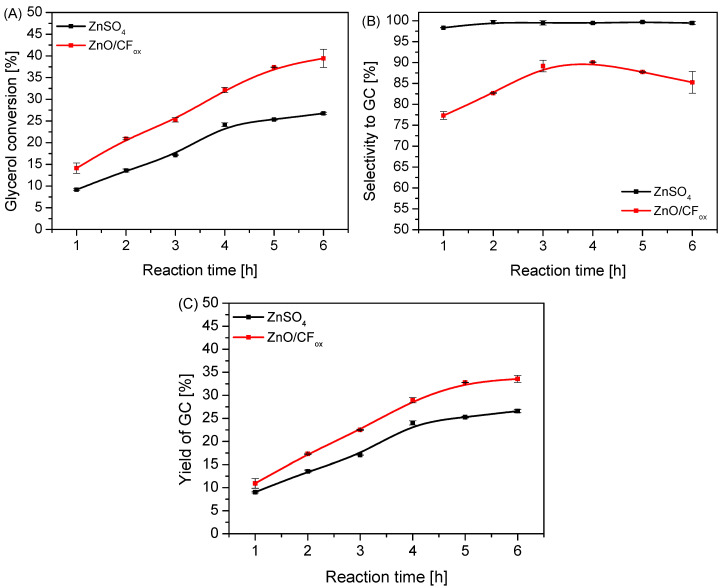
A comparison of the catalytic performances of the heterogeneous ZnO/CF_ox_ and homogeneous ZnSO_4_ catalysts in urea glycerolysis expressed as glycerol conversion (**A**) selectivity to GC (**B**), and yield of GC (**C**); catalyst loading = 3 wt.% in both cases, temp. = 140 °C, Gly:U molar ratio = 1:1, Ar flowing through the reactor.

Figure 9 presents a comparison of the catalytic performances of ZnO/CF_ox_ and a typical homogeneous zinc-based catalyst, i.e., ZnSO_4_. Surprisingly, the ZnO/CF_ox_ sample converted glycerol much more efficiently than ZnSO_4_, reaching a 5% higher X_Gly_ after just 1 h of reaction (Figure 9A). The conversion of glycerol increased over time for both samples; however, ZnO/CF_ox_ was significantly more active in the process, finally showing X_Gly_ of about 40% after 6 h. This suggests good dispersion of ZnO on the CF_ox_ support. On the other hand, considering the results of selectivity to GC (Figure 9B), it is clear that using ZnO/CF_ox_ as a catalyst promoted the formation of a quite significant amount of intermediate and side products (see Figure S2). This was seen particularly clearly in the first hours of the process, when S_GC_ was in the range of 78 and 83%. S_GC_ increased slightly over time; however, it did not exceed 90% when using ZnO/CF_ox_ as a catalyst. On the other hand, ZnSO_4_ worked selectively to glycerol carbonate, and the S_GC_ parameter was almost 100% throughout the reaction. The highly selective performance of the homogeneous ZnSO_4_ catalyst under the conditions used was probably due to the fact that every single catalytic entity could act as a single active site, which is a common advantage of homogeneous systems over heterogeneous ones [49]. Surprisingly, considering the results of GC yields presented in Figure 9C, it can be seen that using the ZnO/CF_ox_ sample allowed us to obtain slightly higher Y_GC_ values than those achieved in the reaction with ZnSO_4_.

**Figure 10:**
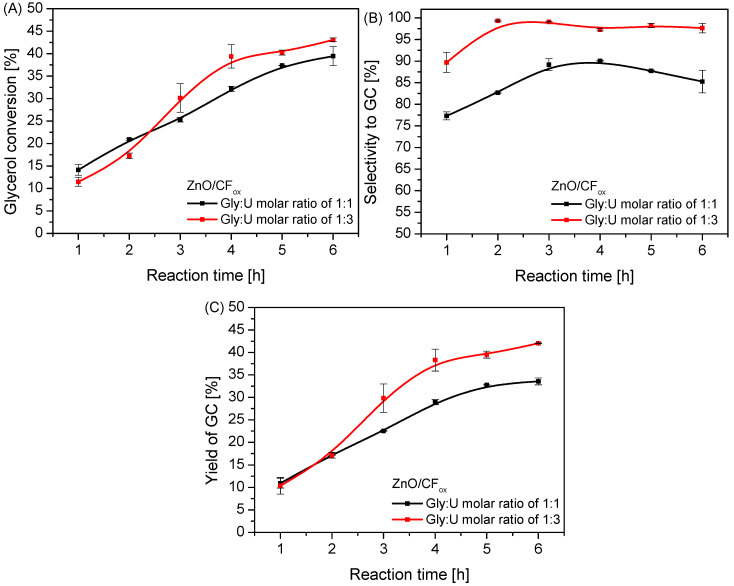
The influence of a Gly:U molar ratio on the process of urea glycerolysis (glycerol conversion (**A**), selectivity to GC (**B**), and yield of GC (**C**)) over the ZnO/CF_ox_ catalyst; temp. = 140 °C, Ar flowing through the reactor.

In order to investigate the effect of different reaction conditions (i.e., Gly:U molar ratio and a reaction temperature) on the urea glycerolysis, additional catalytic tests were carried out over ZnO/CF_ox_. As can be seen in Figure 10, using a Gly:U molar ratio of 1:3 resulted in improved glycerol conversion compared to the value obtained in the reaction performed at an equimolar ratio of the reagents; however, this effect was observed only after 2.5 h of processing. Finally, the achieved X_Gly_ using a 1:3 Gly:U ratio was about 43% after 6 h. Importantly, there were substantial changes in the selectivity to GC when the reaction was performed at various glycerol-to-urea molar ratios, and the process was more selective when using a Gly:U ratio of 1:3, i.e., the use of urea excess resulted in a lower selectivity to GU, higher selectivity to GC, and a lower selectivity to by-products compared to the reaction using a Gly:U molar ratio of 1:1 (compare Figure 10B and Figure S3). It can be assumed that this could be associated with shifting the reaction equilibrium towards the formation of glycerol carbonate due to the excess of urea. Similar results have also been reported by Mallesham et al. [6]. Consequently, the use of Gly:U molar ratio of 1:3 resulted in an almost 10% higher yield of GC after 6 h of processing compared to the reaction using a Gly:U molar ratio of 1:1, as observed in Figure 10C. 

**Figure 11:**
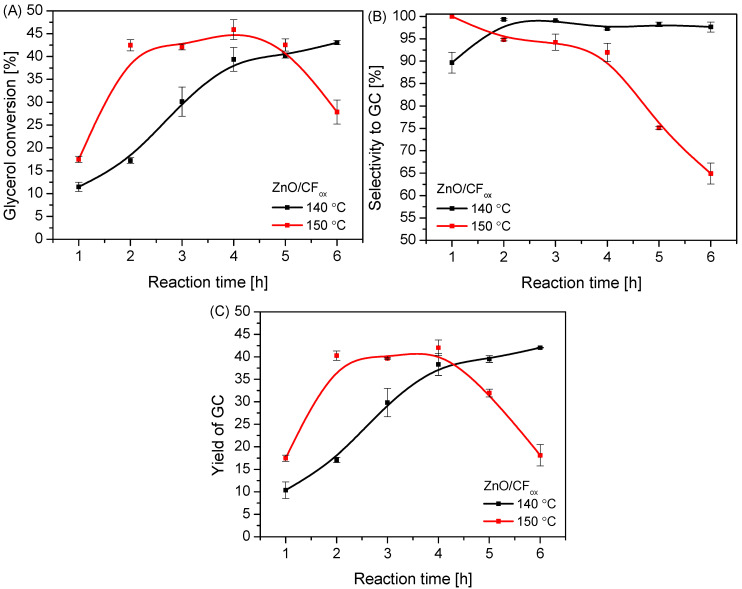
The influence of reaction temperature on the process of urea glycerolysis (glycerol conversion (**A**), selectivity to GC (**B**), and yield of GC (**C**)) over the ZnO/CF_ox_ catalyst; Gly:U molar ratio—1:3, Ar flowing through the reactor.

**Figure 12:**
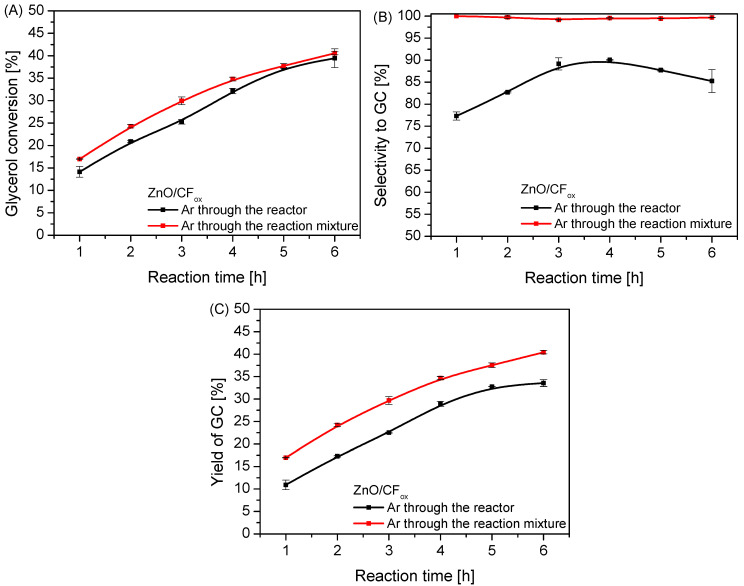
The results of urea glycerolysis (glycerol conversion (**A**), selectivity to GC (**B**), and yield of GC (**C**)) over ZnO/CF_ox_ performed in two variants of Ar flow; temp. = 140 °C, Gly:U molar ratio = 1:1.

The data presented in Figure 11 show that temperature also had a significant impact on the reaction; specifically, using a temperature of 150 °C resulted in a significantly higher conversion of glycerol compared to a temperature of 140 °C, which was observed within 4 h of processing (Figure 11A). Interestingly, at this time, a slight decrease in the S_GC_ parameter over time was observed (in contrast to the reaction at 140 °C), indicating that the more drastic conditions (i.e., high temperature combined with excess urea in the reaction medium) promoted the occurrence of side reactions and further reaction of GC to by-products (see also Figure S4B). The same was observed by Mallesham et al. [6]. Surprisingly, after 4 h of reaction at 150 °C, X_Gly_ and S_GC_ decreased rapidly. This was probably due to the ineffective removal of ammonia from the reaction medium (Ar was flowing through the reactor, see also Figure 12), causing a reverse reaction, as presented in Figure 1. It is also worth mentioning that the higher the glycerol conversion to GC (as observed at 150 °C), the higher the concentration of NH_3_ produced. This was probably the reason for the decrease in X_Gly_, as well as the increase in selectivity to by-products at higher temperatures. The obtained profiles of the GC yield vs. time (Figure 11C) were similar to those of X_Gly_ vs. time. 

In order to effectively flush out the ammonia from the reaction system and thus minimize the undesirable reverse reactions that occurred during the process, an inert gas (Ar) was used and passed through the reactor (i.e., above the surface of the reaction mixture) or directly through the reaction mixture. The obtained results are collected in Figure 12. As can be seen in Figure 12A, the method of passing Ar through the system had a rather small impact on the glycerol conversion. Nevertheless, the value of X_Gly_ obtained in the process using the “through-mixture” mode was slightly increased compared to when using the “through-reactor” mode, especially at the beginning of the process. Interestingly, the method of passing Ar through the system significantly affected the selectivity to glycerol carbonate (Figure 12B) and resulted in S_GC_ increasing to 100%, unchanging over time, when using the “through-mixture” mode. This was most likely due to the efficient forced removal of ammonia from the reaction mixture, causing a shift in the chemical equilibrium towards glycerol carbonate and limiting the formation of by-products [4] (see also the results of selectivity to intermediate product and by-products provided in Figure S5). Similar conclusions were also drawn by Wang et al. [7], who in turn used high vacuum to obtain a satisfactory GC yield. Importantly, changing the method of ammonia removal from the reaction medium from the “through-reactor” to the “through-mixture” mode resulted in a significant increase in the yield of the most desired product, i.e., glycerol carbonate, as seen in Figure 12C. 

Table 3 shows the results achieved in the presence of the best-working catalyst obtained in this study, i.e., ZnO/CF_ox_, and other zinc-containing systems described in the literature. 

As can be observed, the Zn-based catalysts reported in the literature gave similar or higher yields of GC compared to the sample used in our experiment. However, considering the selectivity to the most desirable product (i.e., glycerol carbonate; GC), it is obvious that our catalyst was one of the samples that worked most selectively to GC during the reaction. Although the Zn/MCM-41(im) and ZnCl_2_ samples also showed almost complete selectivity to GC, these catalysts required aggressive conditions (i.e., higher temperature, the use of vacuum, or a large amount of catalyst) to work well in urea glycerolysis. In turn, our experiments were performed under relatively mild conditions, and this may be the reason for the rather moderate catalytic performance of the produced samples. Nevertheless, the obtained results are still quite attractive compared to those obtained in the presence of other zinc-containing systems, especially with regard to the limited production of by-products and a viable and affordable method of GC synthesis.

## 3. Materials and Methods

### 3.1. Preparation of the Catalysts

Metal oxides supported on carbon fibers were produced and applied as catalysts for urea glycerolysis.

The synthesis of the initial carbon fibers (CF) was performed in a horizontal tube furnace by a catalytic chemical vapor deposition (CCVD) method using liquefied petroleum gas (LPG) as a carbon source and metallic nickel as a growth catalyst [51,52,53]. Briefly, 0.1 g of NiO was placed in a quartz boat and heated to 550 °C under argon flow (100 cm^3^/min; heating rate of 10 °/min). Upon reaching the desired temperature, the reduction of NiO to metallic Ni was performed by treating the sample under 20%H_2_/Ar flow for 2 h. Subsequently, the oven temperature was increased to 600 °C, and LPG was passed through the reactor. The CCVD process was carried out for 4 h using a 50%LPG/50%H_2_ gaseous mixture (total flow rate of 100 cm^3^/min) for carbon growth. To remove the residual metal catalyst, the obtained carbon sample was boiled with a 21% HCl solution under reflux conditions for 2 h. Afterward, it was filtered off and washed with hot distilled water until the pH of the filtrate was 7. Finally, the produced material was dried overnight at 110 °C and sieved to a particle size of ≤0.4 mm. 

The obtained CF sample was used to support various metal oxides, i.e., Me_x_O_y_, including Zn, Ba, Cr, and Mg oxides. The deposition of the active phase on the CF was carried out by applying an incipient wetness impregnation technique and mixing the support with aqueous solutions of the respective metal nitrates (using the amounts suitable for obtaining 10 wt.% Me_x_O_y_ loading). After a 24 h impregnation step at ambient temperature, the samples were dried overnight at 110 °C and sieved to a particle size of ≤0.4 mm. Afterward, they were thermally treated at 600 °C under Ar flow (30 cm^3^/min) for 1 h to decompose the nitrates. Finally, the samples were oxidized at 300 °C for 3 h under air/Ar and pure air flow (total flow rate of 20 cm^3^/min in both cases). The obtained materials were labeled according to the scheme Me_x_O_y_/CF_ox_, where Me = Zn, Ba, Cr, or Mg. As in the process, in addition to metals, CF was also oxidized, the symbol CF_ox_ was used instead of CF in the above formula. In order to obtain a sample whose structure and properties would be similar to those of the CF_ox_ support, the initial CF sample was also oxidized (using the same reaction conditions as described above) and designated as CF_ini_ox_.

### 3.2. Characterization of the Samples

The morphological features of the initial CF, CF_ini_ox_ support, and Me_x_O_y_/CF_ox_ catalysts were studied using a scanning electron microscope ZEISS EVO 40. The textural parameters of the samples were determined by nitrogen adsorption/desorption measurements performed at -196 °C and using a Quantachrome Autosorb IQ apparatus. The BET equation was used to calculate the apparent surface area (S_BET_) of the samples, while the t-plot method was applied to determine the micropore volumes (V_micro_) and the external surface areas (S_ext_) of the materials. The total pore volumes (V_tot_) of the samples were calculated from the amount of N_2_ adsorbed at a relative pressure close to 1. Before the textural analysis, the samples were degassed under vacuum at 150 °C for 12 h. Thermogravimetric (TG) analysis was performed by applying a Setaram Setsys 1200 thermal analyzer working in an air flow and at a temperature range of 20–1000 °C (heating rate of 10 °C/min). X-ray diffraction (XRD) measurements were carried out using a Bruker AXS D8 Advance diffractometer equipped with a Johansson monochromator (λCu Kα1 = 1.5406 Å) and a silicon strip detector LynxEye. X-ray photoelectron spectroscopy (XPS) studies were performed using a SPECS Phoibos 150 UHV-XPS spectrometer equipped with a Phoibos HSA3500 analyzer operating in a Fixed Analyzer Transmission (FAT) mode with a pass energy of 20 eV for core-level peaks. The acquired XPS spectra were processed with CasaXPS software (version 2.3.25PR1.0) using a Shirley-type background. The C 1s peak at 284.5 eV was applied as an internal standard and fitted with an asymmetric LF line shape. The other peaks in the C 1s and Zn 2p regions were constrained with a mixed Gaussian–Lorentzian (GL) function.

### 3.3. Catalytic Tests

The reaction of glycerol (Gly) with urea (U) was performed in a round-bottom flask equipped with a magnetic stirrer, thermocouple, condenser, and an inert gas (Ar) supply. The reagents, at a Gly:U molar ratio of 1:1 or 1:3, were placed in the reactor and homogenized. After heating the mixture to the desired temperature (140 or 150 °C), a catalyst (3 wt.% with respect to the glycerol mass) was added to the flask. The reaction was carried out under Ar flow, which was passed either through the reactor or directly through the reaction mixture. To monitor the progress of the process, samples of the reaction mixture were taken periodically and analyzed using a gas chromatograph (SRI 8610C) equipped with an MXT-5 capillary column, flame ionization detector (FID), and a split injector. Helium was used as a carrier gas, and the analyses were performed isothermally at 160 °C. The catalytic activities of the tested samples were expressed as conversion of glycerol (X_Gly_), yield of glycerol carbonate (Y_GC_), and selectivity to GC (S_GC_). To determine the reproducibility of analytical data, the standard deviation values (SD) of these parameters were also calculated for each sample. Selectivities to glycerol urethane or by-products (S_GU_ and S_B-Py_) were also calculated. The obtained data are presented in the Supplementary Materials. For the sake of comparison, reactions without a catalyst and in the presence of a homogeneous ZnSO_4_ catalyst were also performed. 

## 4. Conclusions

A series of metal oxides supported on modified carbon fibers were developed and used as catalysts for the conversion of glycerol to glycerol carbonate under ambient pressure. Among the prepared systems, ZnO/CF_ox_ and MgO/CF_ox_ gave the most promising catalytic results, which was probably due to the presence of well-balanced acid–base properties of these samples. The reaction temperature, molar ratio of the reagents, and the applied mode of the inert gas flow significantly affected the conversion of glycerol and/or selectivity to glycerol carbonate. Using an increased amount of urea (3 moles instead of 1 per 1 mole of glycerol) slightly improved the conversion of glycerol, whereas the selectivity to glycerol carbonate increased considerably (to almost 100%) under these conditions. Interestingly, the same effect was obtained when Ar was passed through the reaction mixture instead of passing Ar through the reactor (above the surface of the reaction mixture). On the other hand, an increase in the reaction temperature resulted in an improvement in glycerol conversion; however, at the same time, this negatively affected the selectivity to glycerol carbonate. Under the best reaction conditions used, the high yield of glycerol carbonate of about 40%, together with ~100% selectivity to GC, was obtained over the ZnO/CF_ox_ catalyst.

## Figures and Tables

**Table 1:** Textural parameters of the prepared CF, CF_ini_ox_, and CF_ox_-supported samples.

Sample	S_BET_ [m^2^/g]	S_ext_ [m^2^/g]	V_tot_ [cm^3^/g]	V_micro_ [cm^3^/g]
CF	259	187	1.22	0.04
CF_ini_ox_	267	193	0.97	0.04
Cr_2_O_3_/CF_ox_	236	159	0.70	0.04
BaO/CF_ox_	214	214	1.02	0.00
MgO/CF_ox_	213	163	0.86	0.03
ZnO/CF_ox_	238	170	0.79	0.04

**Table 2:** The contents of elements measured for the ZnO/CF_ox_ sample by the XPS technique.

Sample	C [wt.%]	O [wt.%]	Zn [wt.%]	ZnO in ZnO/CF_ox_ * [wt.%]
ZnO/CF_ox_	87.1	5.4	7.5	9.3

* ZnO content calculated based on the amount of zinc from XPS.

**Table 3:** A comparison of the catalytic performances of different Zn-based systems in urea glycerolysis.

Sample	Reaction Conditions	X_Gly_ [%]	Y_GC_ [%]	S_GC_ [%]	Reference
ZnO/CF_ox_	140 °C; Gly:U molar ratio of 1:1; 6 h; Ar flow (20 mL/min); Catalyst loading of 3%wt. (with respect to glycerol mass)	40.5	40.4	99.7	This work
ZnO	130 °C; Gly:U molar ratio of 1:1; 3 h; reaction pressure of 3 kPa; Catalyst loading of 5.4%wt. (with respect to glycerol mass)	61.0	42.0	69.0	[18]
2.5 wt.%Au/ZnO	150 °C; Gly:U molar ratio of 1:1.5; 4 h; N_2_ flow; Catalyst loading of ~2%wt. (with respect to glycerol mass)	88.0	56.0	49.0	[29]
Zn_2_CrO	140 °C; Gly:U molar ratio of 1:1; 3 h; reaction pressure of 3 kPa; Catalyst loading of ~5%wt. (with respect to glycerol mass)	76.0	57.0	74.0	[16]
Zn/MCM-41(im)	145 °C; Gly:U molar ratio of 1:1; 5 h; N_2_ flow; Catalyst loading of 5%wt. (with respect to glycerol mass)	75.0	73.0	98.0	[32]
ZnCl_2_	150 °C; Gly:U molar ratio of 1:1; 2 h; reaction pressure of 2.67 kPa; Catalyst loading of 2 mol% (with respect to glycerol mass)	80.4	80.2	99.7	[11]
Zn(OAc)_2_·2H_2_O	150 °C; Gly:U molar ratio of 1:1; 2 h; reaction pressure of 2.67 kPa; Catalyst loading of 2 mol% (with respect to glycerol)	67.2	44.3	66.0	[11]
50%-Zn_7_Al_3_O_x_/ARM	140 °C; Gly:U molar ratio of 1:1; 5 h; reaction pressure of 3 kPa; Catalyst loading of 5%wt. (with respect to glycerol)	69.0	58.1	84.2	[50]

## Data Availability

The data presented in this study are available on request from the corresponding authors.

## Supplementary Materials

The following supporting information can be downloaded at: https://www.mdpi.com/article/10.3390/molecules28186534/s1, Figure S1. The results of selectivity to different products obtained in the blank test and in the reaction over CF_ini_ox_ sample and CF_ox_-supported catalysts (GU – glycerol urethane; GC – glycerol carbonate); Figure S2. The results of selectivity to A) glycerol urethane (GU) and B) by-products obtained for the homogeneous and CF_ox_-supported ZnO catalysts vs. time; Figure S3. The results of selectivity to (A) glycerol urethane (GU) and (B) by-products obtained over the CF_ox_-supported ZnO catalyst using different glycerol to urea (Gly:U) molar ratios; Figure S4. The results of selectivity to (A) glycerol urethane (GU) and (B) by-products obtained over the CF_ox_-supported ZnO catalyst at different temperatures; Figure S5. The results of selectivity to (A) glycerol urethane (GU) and (B) by-products obtained over the CF_ox_-supported ZnO catalyst using different reaction set-ups.
