# Supplementary material for: A Green Approach to Obtaining Glycerol Carbonate by Urea Glycerolysis Using Carbon-Supported Metal Oxide Catalysts
Source: Molecules. 2023 Sep 9;28(18):6534. doi: 10.3390/molecules28186534 (PMC10538211; doi:10.3390/molecules28186534)
Supplement: Supplementary file 1 [file molecules-28-06534-s001.zip › molecules-2561640-supplementary.pdf]

SUPPLEMENTARY MATERIAL

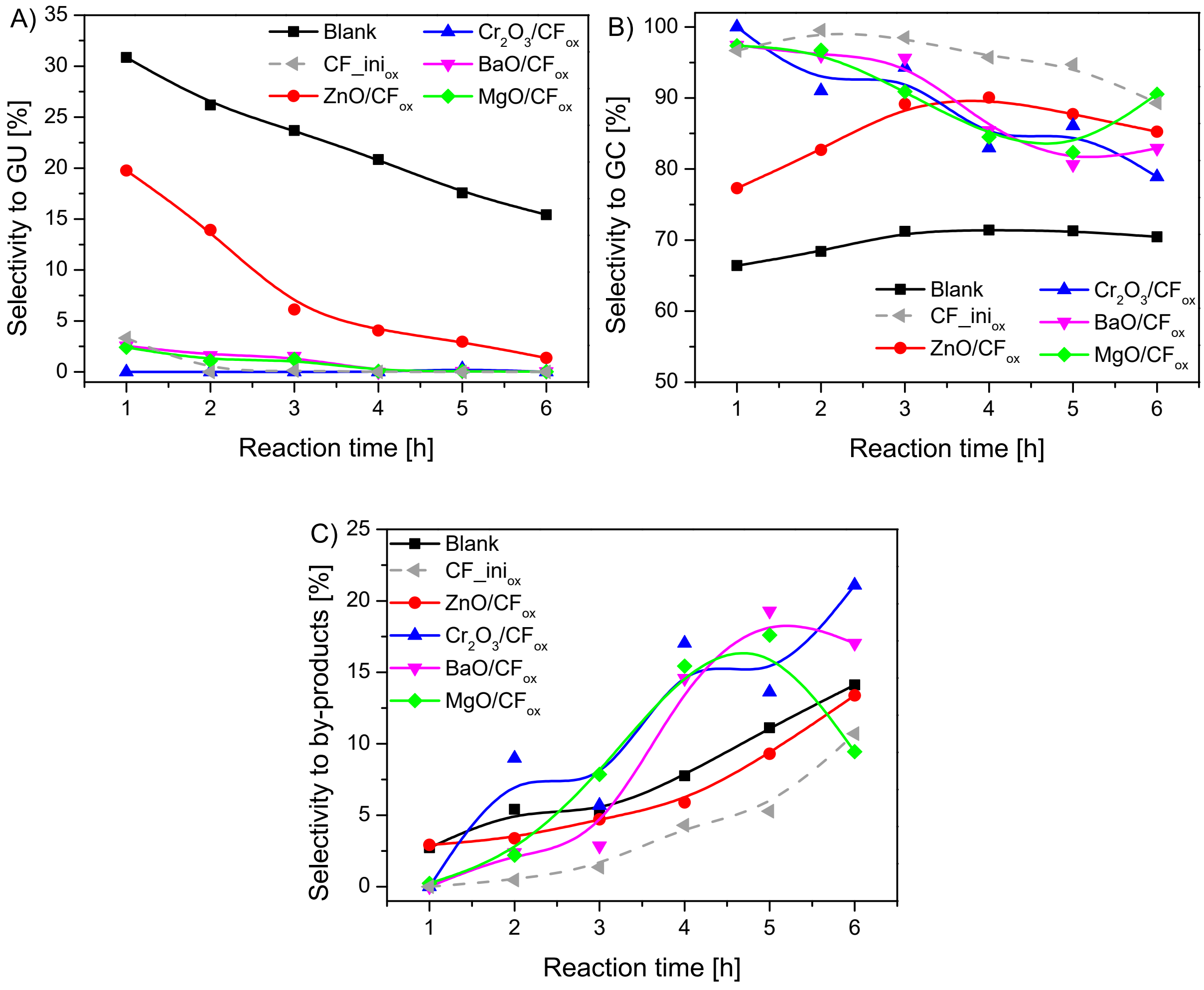

Figure S1. The results of selectivity to different products obtained in the blank test and in the reaction over CF_ini$_{ox}$ sample and CF$_{ox}$-supported catalysts (GU – glycerol urethane; GC – glycerol carbonate)

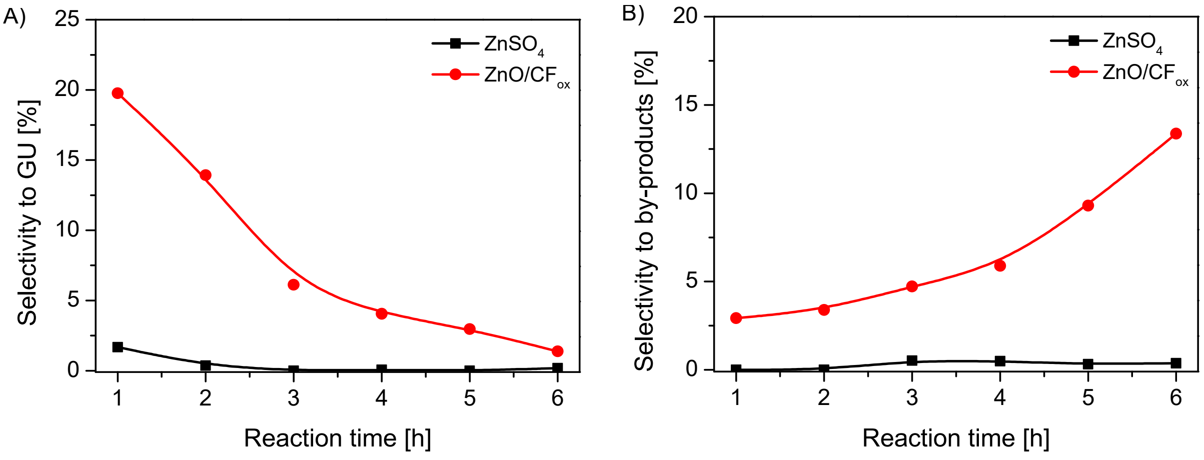

Figure S2. The results of selectivity to A) glycerol urethane (GU) and B) by-products obtained for the homogeneous and CF$_{ox}$-supported ZnO catalysts vs. time

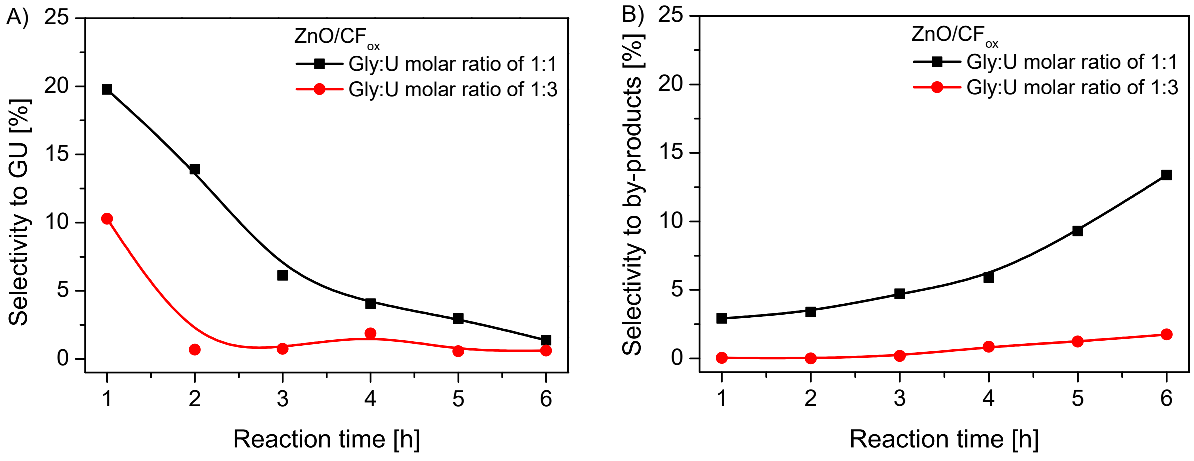

Figure S3. The results of selectivity to A) glycerol urethane (GU) and B) by-products obtained over the CF_{ox}-supported ZnO catalyst using different glycerol to urea (Gly:U) molar ratios

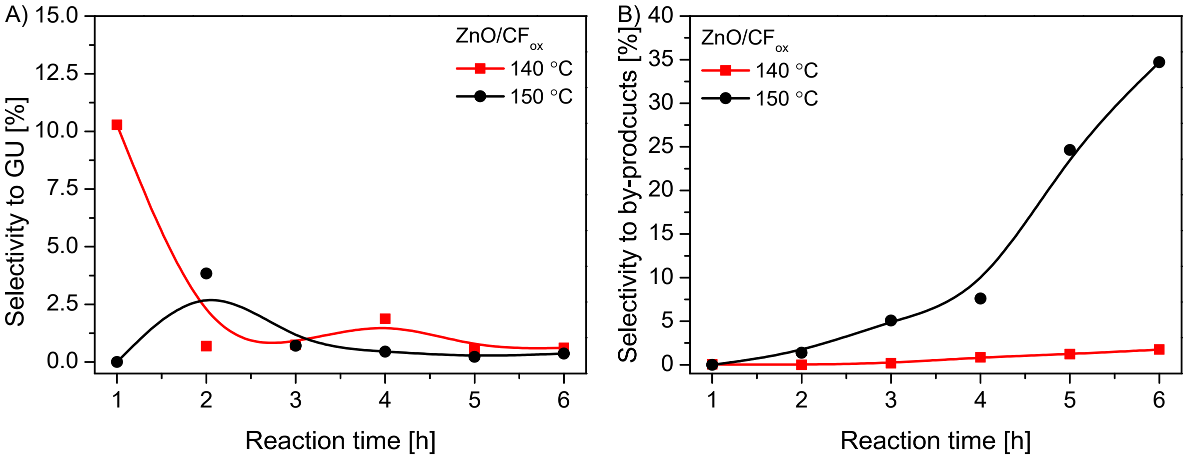

Figure S4. The results of selectivity to A) glycerol urethane (GU) and B) by-products obtained over the CF_{ox}-supported ZnO catalyst at different temperatures

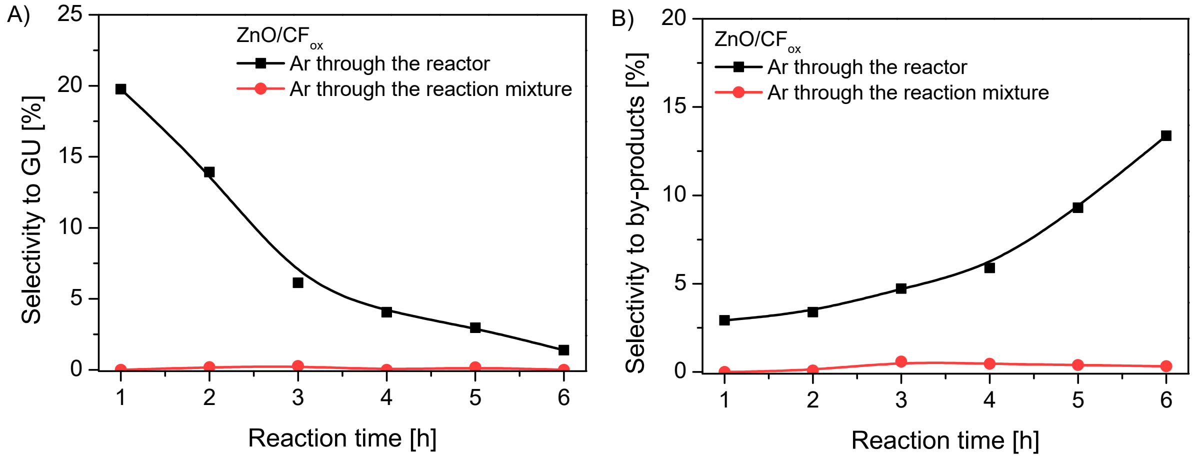

Figure S5. The results of selectivity to A) glycerol urethane (GU) and B) by-products obtained over the CF_{ox}-supported ZnO catalyst using different reaction set-ups
